# Obesity Potentiates Esophageal Squamous Cell Carcinoma Growth and Invasion by AMPK-YAP Pathway

**DOI:** 10.1155/2020/6765474

**Published:** 2020-12-10

**Authors:** Jia-Huang Liu, Qi-Fei Wu, Jun-Ke Fu, Xiang-Ming Che, Hai-Jun Li

**Affiliations:** ^1^Department of Thoracic Surgery, The First Affiliated Hospital of Xi'an Jiaotong University, Xi'an 710061, China; ^2^Department of General Surgery, The First Affiliated Hospital of Xi'an Jiaotong University, Xi'an 710061, China

## Abstract

Obesity could increase the risk of esophageal squamous cell carcinoma (ESCC) and affect its growth and progression, but the mechanical links are unclear. The objective of the study was to explore the impact of obesity on ESCC growth and progression utilizing in vivo trials and cell experiments in vitro. Diet-induced obese and lean nude mice were inoculated with TE-1 cells, then studied for 4 weeks. Serum glucose, insulin, leptin, and visfatin levels were assayed. Sera of nude mice were obtained and then utilized to culture TE-1. MTT, migration and invasion assays, RT-PCR, and Western blotting were used to analyze endocrine effect of obesity on cell proliferation, migration, invasion, and related genes expression of TE-1. Obese nude mice bore larger tumor xenografts than lean animals, and were hyperglycemic and hyperinsulinemic with an elevated level of leptin and visfatin in sera, and also were accompanied by a fatty liver. As for the subcutaneous tumor xenograft model, tumors were more aggressive in obese nude mice than lean animals. Tumor weight correlated positively with mouse body weight, liver weight of mice, serum glucose, HOMA-IR, leptin, and visfatin. Obesity prompted significant TE-1 cell proliferation, migration, and invasion by endocrine mechanisms and impacted target genes. The expression of AMPK and p-AMPK protein decreased significantly (*P* < 0.05); MMP9, total YAP, p-YAP, and nonphosphorylated YAP protein increased significantly (*P* < 0.05) in the cells cultured with conditioned media and xenograft tumor from the obese group; the mRNA expression of AMPK decreased significantly (*P* < 0.05); YAP and MMP9 mRNA expression increased significantly (*P* < 0.05) in the cells exposed to conditioned media from the obese group. In conclusion, the altered adipokine milieu and metabolites in the context of obesity may promote ESCC growth in vivo; affect proliferation, migration, and invasion of ESCC cells in vitro; and regulate MMP9 and AMPK-YAP signaling pathway through complex effects including the endocrine effect.

## 1. Introduction

Esophageal carcinoma (EC) is one of the most common gastrointestinal malignancies in the world. ESCC and esophageal adenocarcinoma (EAC) account for more than 95% of EC. ESCC is more predominant in South America, Africa, and Asia [1] and acts as the leading cause of cancer-related death in China [2]. ESCC has a poor prognosis and high mortality rate, most of the patients are diagnosed in the middle or advanced stage, and the general outcome remains very poor for overall 5-year survival rates of about 10% [3]. Exploring the mechanisms of ESCC growth and development is therefore critical to identify novel therapeutic targets for desperately needed adjuvant treatment.

Obesity is a worldwide health issue, and its prevalence has doubled since 1980 in more than 70 countries and continuously increased in most countries [4]. Obesity is proved to be a risk factor for developing a variety of malignancies, including EAC, cardia gastric cancer, and breast cancer [5–7]. The relationship between obesity and ESCC is very complicated, and the obesity paradox in cancer that obesity may promote cancer development, but not correlate with overall survival of these patients, exists, especially for ESCC [5, 8], and a consensus is very hard to reach. But the mainstream opinion is that obesity, especially abdominal obesity, may be correlated with an increased risk for ESCC [9, 10]. Dysregulated metabolism that stems from obesity, including hyperglycemia, insulin resistance, and dyslipidemia, could further affect tumor growth and development. Obesity also has cumulative white adipose tissue (WAT). WAT acts as an active endocrine organ and secretes a number of soluble mediators named as adipokines which play a dynamic role in regulating metabolism, inflammation, immunity, and so on; the changed adipokine milieu of obesity also impacts on potentiating the development of diseases such as diabetes, cardiovascular disease, and cancer [11, 12]. Adipokines such as leptin and other growth factors secreted on the background of obesity may influence cancer cell survival and solid tumor growth [11]. Leptin positively correlates with adipose storage and nutritional state and plays an important role in energy balance and appetite control and is also known as a potential mediator of obesity-associated cancers [13]. The new adipokine, visfatin, also mainly secreted by visceral fat, elevates with the increase of obesity [14] and coordinates with glucose and lipid metabolism. Visfatin may also contribute to being a significant mechanistic link in the network of factors influencing obesity-related tumor growth [15].

Yes-associated protein (YAP), a transcriptional coactivator, functions downstream of the Hippo signaling pathway and regulates multiple cellular processes. Dysregulation of the Hippo-YAP pathway existed frequently in human cancers, including oral squamous cell carcinoma [16], gastric cancer [17], and ESCC [18]. YAP shuttles between the cytoplasm and nucleus, where it interacts with transcription factors, particularly TEA domain (TEAD) family members, then prompts proproliferative and antiapoptotic gene expression [19]. When the upstream Hippo kinase receives an extracellular signal to inhibit cell growth, YAP is phosphorylated and inactivated in the cytoplasm, then gets subsequent ubiquitin-mediated proteasomal degradation. By contrast, when the kinase gets a growth prompting signal, hypophosphorylated YAP translocates into the nucleus and induces the expression of target proproliferative and antiapoptotic genes. Adenosine monophosphate-activated protein kinase (AMPK) is a highly conserved serine/threonine protein kinase consisting of a catalytic *α*-subunit and regulatory *β*- and *γ*-subunit. The *α*-subunit contains a conserved threonine residue (Thr172) which was phosphorylated by upstream protein kinases leading to AMPK activation [20]. AMPK is a key cellular energy sensor activated by increasing AMP levels, coordinates cell growth with energy availability, and is also inactivated in an obese state. Cellular energy stress induces YAP phosphorylation, in part due to AMPK-dependent Lats activation, thereby inhibiting YAP activity. In addition, AMPK directly phosphorylated YAP S94, a residue essential interacting with TEAD, thus disrupting the YAP-TEAD interaction [21]. Therefore, the AMPK-YAP pathway links cellular energy status such as obesity to tumorigenesis and may play a significant role in the growth and development of obesity-related cancers. EC is available for early lymphatic and haematogenous dissemination, and that is closely related to tumor invasion and metastasis [22]. Matrix metalloproteinases (MMPs) function as proteases for proteolytic cleavage of the extracellular matrix (ECM), necessary for tumor invasion and metastasis. MMP2 and MMP9, as important members of the gelatinase family of MMPs which degraded type IV collagen, the principal component of the basement membrane, cause increased tumor metastasis [23, 24]. In particular, upregulated MMP9 is related to a poorer prognosis in a number of malignant tumors including ESCC and EAC [25–27].

Very little fundamental research has investigated the mechanisms by which obesity affects ESCC growth and development, and no suitable animal models about that exist currently. Therefore, an in vivo animal model and cell experiments in vitro were adopted in the study to explore the impact of obesity on ESCC growth and development and the related molecular mechanism.

## 2. Materials and Methods

### 2.1. Animals and Cell Culture

Animal experiments were approved by the Xi'an Jiaotong University Institutional Animal Care and Use Committee. Four- to five-week-old male nude mice obtained from Slac Laboratory animal (Shanghai, China) were housed in standard conditions, then divided into two groups which were fed a high-fat diet (35.0% fat, 26.0% carbohydrate, and 26.0% protein) and normal chow (4.3% fat, 67.3% carbohydrate, and 19.2% protein) [28] for 8 weeks, respectively. TE-1, kyse150, and Eca109 as typical ESCC cell lines were conferred from the experiment platform of Xi'an Jiaotong University or purchased from the cell bank of the Chinese Academy of Sciences, Shanghai, China. All cancer cells were cultured in a Roswell Park Memorial Institute-1640 (RPMI-1640) medium (Cellgro, Herndon, VA) and supplemented with 10% (*v*/*v*) heat-inactivated fetal bovine serum (Valley Biomedical, Winchester, VA), penicillin (100 U/mL), and streptomycin (100 mg/mL). Cells were maintained in a 37°C humidified incubator supplying 5% CO_2_. TE-1 was chosen for further trials depending on the protein expression of MMP9.

### 2.2. *In Vivo* ESCC Model

After 8 weeks, mice fed normal chow were defined as “lean,” and mice consuming high-fat diet and collected by the criterion that the body weight exceeded the mean plus 2-fold standard deviation (SD) of these lean nude mice were defined as “obese,” the animals left consuming the high-fat diet were referred to as “nonobese.” All mice were inoculated subcutaneously with 2.0 × 10^6^ TE-1 cells into the right flank and monitored daily; then, those chosen nude mice were maintained on normal or high-fat diet for another 4 weeks, respectively. Tumor volume was assessed by gauging the length and width of the tumor with calipers as 1/2 (length × width^2^), once tumors became palpable. After 4 weeks of injected tumor cell growth, all mice were sacrificed after anesthesia, and blood drawn from the retroorbital venous plexus was preserved for assessment of metabolites or adipokines. Tumors were carefully dissected, weighed, and then preserved for further analysis. Laparotomy was carried out to detect the existence of metastasis; then, the liver was resected and weighed and also preserved for oil red O staining.

### 2.3. Serum Assays

An enzyme-linked immunosorbent assay (ELISA) was utilized to determine the serum level of insulin, leptin, and visfatin following the manufacturer's instructions. All of the ELISA kits were purchased from Proteintech Group (Rosemont, USA). Serum glucose was assessed by a colorimetric assay.

### 2.4. Cellular Proliferation Assay

Cellular proliferation in vitro was estimated by the MTT assay. After exposure to conditioned media or RPMI-1640, TE-1 cells were incubated with 0.5% MTT solution for another 4 h; then, 150 *μ*L of DMSO was introduced to solubilize the MTT tetrazolium crystal; optical density of them was determined at 490 nm by a Benchmark Plus microplate reader (Bio-Rad, Hercules, CA). All trials were repeated three times.

### 2.5. Migration and Invasion Assays

In order to determine the migration and invasion of TE-1 cells in response to exposure to conditioned media, migration and invasion assays were performed according to the instructions. Briefly, 5 × 10^4^ TE-1 cells per chamber were introduced to the top wells of migration chambers or invasion chambers with a matrigel membrane. Conditioned media or serum-free RPMI-1640 was placed into the wells beneath the chambers for 24 h. After removing the cells from the upper surface of the filters, the remnant cells on the lower surface were counted randomly in 10 different high-power fields (magnification, ×200) using a microscope.

### 2.6. RNA Expression Studies

Total RNA was extracted from target cells with exposure to conditioned media or RPMI-1640 for 24 h, with the help of the TRIzol reagent (Invitrogen, San Diego, CA), then reverse transcribed to cDNA with a cDNA Synthesis Kit (Takara Biochemicals, Japan) following the manufacturer's instructions. Expression of AMPK and YAP mRNA was quantified by RT-PCR (Takara Biochemicals, Japan). Transcript levels were normalized to *GAPDH*. Briefly, the reaction was performed utilizing an iCycler (Bio-Rad, Hercules, CA) with the following thermal profile: a preheating step at 95°C for 10 min, 30 repeats of 94.0°C for 30 sec, 57.0°C for 30 sec, and then 72°C for 1 min. These primers' sequence was generated by NCBI Primer-BLAST (Table 1). For validation, each trial was done three times.

### 2.7. Western Blotting Analysis

Target cells exposed to conditioned media or RPMI-1640 for 24 h or target tumor tissues' extracts were analyzed by Western blotting as previously described to determine whether obesity may influence the growth and development of TE-1 [29]. Briefly, 25 *μ*g of extracted protein was resolved by SDS-PAGE and then electroblotted onto nitrocellulose membranes for Western blotting analysis. Blots were probed with recommended diluted primary antibodies overnight at 4°C, followed by incubation with HRP-conjugated secondary antibody for 1 h at room temperature. The membranes were developed with enhanced chemiluminescence (Pierce) by an enhanced chemiluminescence detection system (Amersham Bioscience, Piscataway, NJ, USA). The primary antibodies recognizing AMPK, p-AMPK, YAP, p-YAP, and GAPDH were purchased from Beijing Biosynthesis Biotechnology (China).

### 2.8. Statistical Analysis

Values were expressed as the mean ± SD. Statistical differences were evaluated by one-way analysis of variance (ANOVA) followed by Dunnett's test; correlation analysis was estimated by the Pearson test. Those *P* < 0.05 were considered statistically significant.

## 3. Results

### 3.1. Expression of MMP9 Protein in ESCC Cell Lines

**Figure 1:**
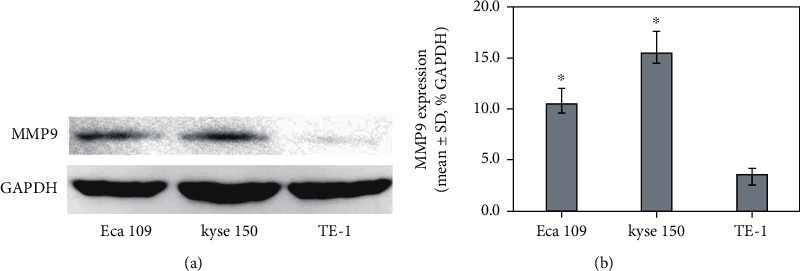
MMP9 protein expression in ESCC cell lines. ^∗^*P* < 0.05 versus TE-1.

One of the key projects of this study was to investigate the invasion of ESCC, and MMP9 protein expression in common ESCC cell lines such as Eca109, kyse150, and TE-1 was analyzed. The result of this study demonstrated that TE-1 had the lowest expression among them (Figure 1) and was chosen as the core cell line for further trials.

### 3.2. Metabolic Changes in Mice

Eight weeks later, 15 nude mice fed the normal chow were defined as “lean,” 14 out of 40 nude mice consuming the high-fat diet were chosen as “obese,” and then, the 26 nude mice left from the high-fat diet group were classified as “nonobese” mice. At the time of inoculating the animals, these obese mice were significantly heavier than lean animals (28.04 ± 0.53 versus 23.84 ± 1.86 g, *P* < 0.001), but no difference was observed between lean and nonobese mice (23.84 ± 1.86 versus 22.01 ± 2.32 g, *P* = 0.42).

### 3.3. Tumor Growth and Development

**Figure 2:**
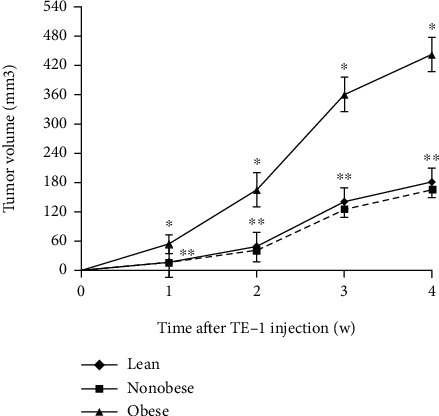
Subcutaneous tumor xenograft growth curve. Tumor xenografts from obese nude mice grew faster than those from lean and nonobese animals within 4 weeks. ^∗^*P* < 0.05 versus lean and nonobese, ^∗∗^*P* > 0.05 versus nonobese.

**Figure 3:**
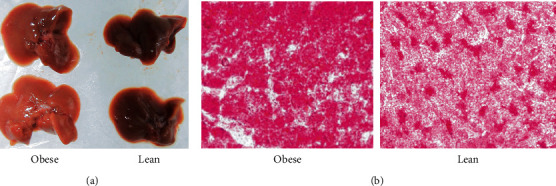
Liver character from obese and lean mice. (a) Unaided observation of the liver showed that the color of the liver became more yellow. (b) Oil red O staining of the liver specimen under a microscope demonstrated that lipid in cytoplasm became red with the help of oil red O, and the fields of the liver specimen from the obese group get more red than that from lean mice. Magnification, ×400.

Injected nude mice were fed the normal or high-fat diet for another 4 weeks. All of these mice were alive, and no metastasis was observed during the experimental time frame. Tumor growth was detected in 93.3% (14/15) of lean mice, in 92.9% (13/14) of obese mice, and in 88.5% (23/26) of nonobese animals. Tumors grew larger and faster in obese nude mice than lean and nonobese nude mice in the 4 weeks (Figure 2). By the end of the animal trial, the body, tumor, and liver weight of mice was collected (Table 2). Tumor weight indicated a strongly positive correlation with body weight of mice (*r*_*s*_ = 0.57, *P* < 0.001). The obese mice also demonstrated a fatty liver (Figure 3), and tumor weight also revealed a strongly positive correlation with liver weight of mice (*r*_*s*_ = 0.62, *P* < 0.001).

### 3.4. Metabolic Parameters and Adipokines

Glucose, insulin, leptin, and visfatin levels in sera, as well as the homeostatic model assessment insulin resistance (HOMA-IR) score, which is a measure of insulin resistance, were collected (Table 3). These obese nude mice demonstrated an altered metabolism milieu such as hyperglycemia, hyperinsulinemia, and insulin resistance and had a higher serum level of leptin and visfatin than lean mice by the end of the animal experiment. Tumor weight correlated positively with glucose (*r*_*s*_ = 0.50, *P* < 0.001), HOMA-IR (*r*_*s*_ = 0.45, *P* = 0.001), leptin (*r*_*s*_ = 0.34, *P* = 0.017), and visfatin (*r*_*s*_ = 0.49, *P* < 0.001) but is not correlated with insulin (*r*_*s*_ = 0.26, *P* = 0.064).

### 3.5. MMP9, AMPK, and YAP Protein Expression in Tumor Tissues

**Figure 4:**
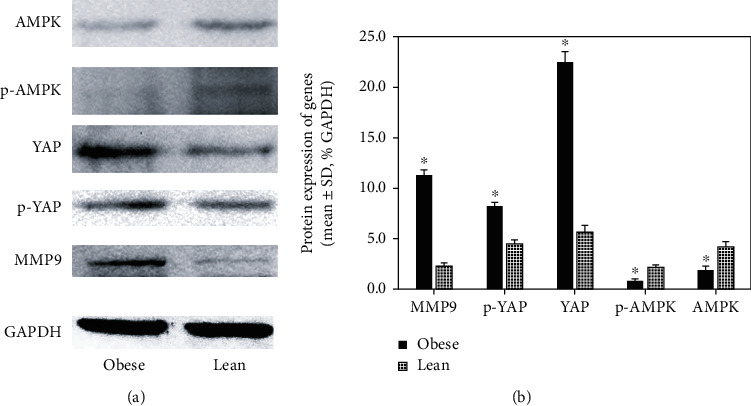
MMP9, AMPK, and YAP protein expression in these subcutaneous tumors was evaluated semiqualitatively. ^∗^*P* < 0.05 versus lean.

Although no local and peritoneal metastasis was detected in the mice, related genes about tumor growth and development were still analyzed in the xenograft tumors by Western blotting. Tumors from obese nude mice had upregulated expression of MMP9, YAP, and p-YAP and lower expression of AMPK and p-AMPK than that from lean mice significantly (Figure 4). Further analysis showed that the active state of YAP, nonphosphorylated YAP, was higher in the tumor from the obese group than the lean group (14.3 ± 0.3 versus 1.1 ± 0.4% GAPDH, *P* < 0.001).

### 3.6. Effects of Obesity on Tumor Cell Proliferation, Migration, and Invasion

**Figure 5:**
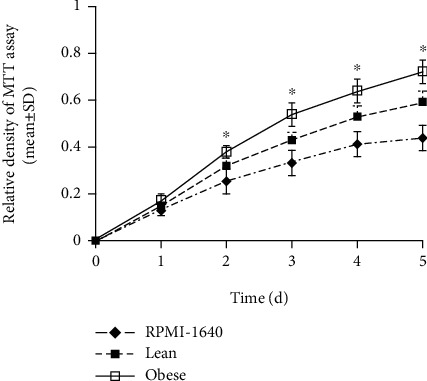
TE-1 cell proliferation in different states. TE-1 cultured with conditioned medium from sera of obese mice grew faster than those from lean or RPMI-1640. ^∗^*P* < 0.05 vs. lean.

In order to detect the role of endocrine effect from obesity, cell experiments in vitro were performed with TE-1 cells under conditioned media. Conditioned media contained 5% sera (*v*/*v*) obtained from obese or lean mice and 95% RPMI-1640. The MTT assay was used to assess TE-1 cell proliferation. These groups included obese, lean, and RPMI-1640 as the control group. After treatment, cell proliferation by the MTT assay was followed over a course of 5 days. The cell growth curve was made, and the results demonstrated that obesity prompted TE-1 cells to grow significantly faster compared to the lean group (*P* < 0.05, Figure 5).

**Figure 6:**
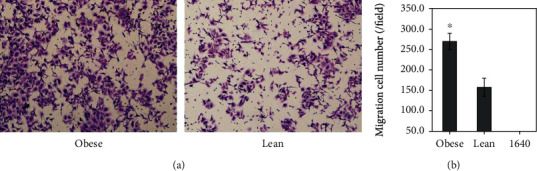
Effects of obesity on TE-1 cell migration. Obesity prompted significantly TE-1 cell migration than the lean (270.1 ± 19.8 versus 158.0 ± 21.6/field, ^∗^*P* < 0.001). Magnification, ×200.

**Figure 7:**
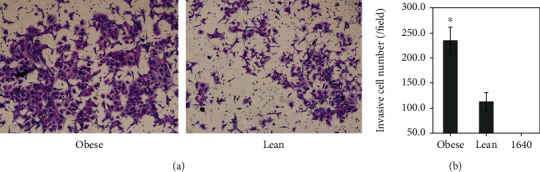
Effects of obesity on TE-1 cell invasion. Obesity accelerated significantly TE-1 cell invasion than the lean (234.0 ± 26.9 versus 112.4 ± 18.9/field, ^∗^*P* < 0.001). Magnification, ×200.

Individual cell migration and invasion is a significant feature of cancer cells to form local or distant metastasis, so a cell migration and invasion assay was performed. The migrating cell quantity of the obese group was significantly larger than that of the lean group in the high-power fields (magnification, ×200) (270.1 ± 19.8 versus 158.0 ± 21.6/field, *P* < 0.001, Figure 6), and no cell migration existed in the RMPI-1640 group. The invasive cell quantity of the obese group was also significantly larger than that of the lean group in the high-power fields (234.0 ± 26.9 versus 112.4 ± 18.9/field, *P* < 0.001, Figure 7). The results showed that obesity functioning as a chemokine induced TE-1 cells traversing the filter and prompted some MMP expression to act upon proteolytic cleavage of the matrigel membrane, then traverse it.

### 3.7. Obesity Promoting Growth and Development of ESCC by MMP9 and AMPK-YAP

**Figure 8:**
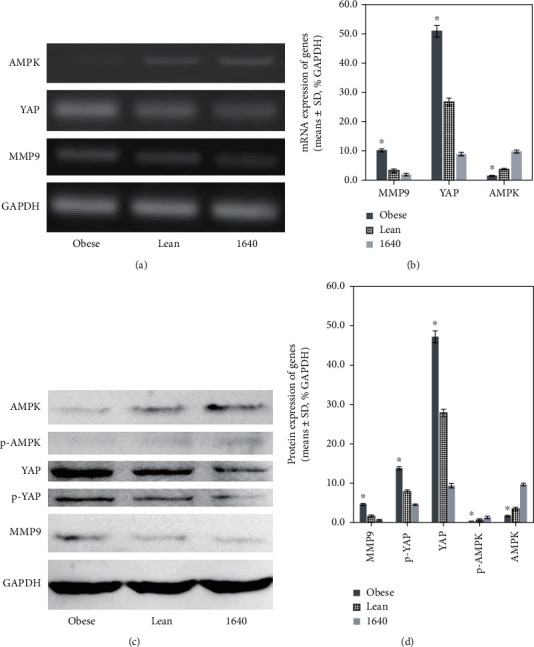
Related mechanism that diet-induced obesity accelerated TE-1 cell growth and progression was dissected. Obesity prompted the growth and development of TE-1 cells by MMP9 and AMPK-YAP signaling pathway at the mRNA level by RT-PCR (a, b) and at the protein level by Western blotting (c, d). ^∗^*P* < 0.05 versus lean.

For validation and further analysis of the endocrine mechanism from obesity, the expression of these related genes MMP9, AMPK, and YAP in the cultured TE-1 cells under different conditions was investigated. mRNA expression of AMPK decreased significantly (*P* < 0.05); YAP and MMP9 mRNA expression elevated significantly (*P* < 0.05) in the cells cultured with conditioned media from the obese group. Protein expression of AMPK and p-AMPK declined significantly (*P* < 0.05); MMP9, total YAP, and p-YAP protein was significantly upregulated (*P* < 0.05) in TE-1 cells in the context of obesity (Figure 8). Further analysis also revealed that nonphosphorylated YAP expression still rose in TE-1 cells cultured with conditioned media from the obese group than that from the lean group (33.4 ± 0.5 versus 20.0 ± 0.9% GAPDH, *P* < 0.001). Therefore, obesity could potentiate TE-1 cell proliferation, migration, and invasion and altered gene expression by endocrine effect.

## 4. Discussion

Obesity confers increased risk for a variety of serious conditions and is increasingly recognized as a cause of preventable cancer risk such as ESCC. The mechanisms linking obesity with ESCC are complicated since lots of biological effects of obesity exist, such as tumor microenvironment, abnormal adipokine secretion, oxidative stress, metabolites, inflammation, immunity, and complex effect [12]. These effects can be categorized into cancer-stromal interaction, paracrine, autocrine, and endocrine mechanism [30]. Altered metabolic activity of visceral AT in obesity may play a fundamental role in the development of these obesity-associated cancers, and AT may influence the progression of ESCC by increased cancer cell growth or invasion and decreased apoptosis [31]. Cancer-stromal interaction driven by cell-cell contact and autocrine, paracrine, and endocrine mechanisms is critical for the growth, invasion, and metastasis of various cancers such as EC [32].

Therefore, we developed modeling ESCC in vivo and cell trials in vitro utilizing the sera of mice to determine this correlation between obesity and ESCC in the study. The results demonstrated that TE-1 xenograft tumors grew larger in obese nude mice than that in lean animals, which strongly demonstrated the positive correlation between obesity and ESCC growth, providing powerful statistic support for the direct effect of obesity on ESCC growth. The results also showed that obesity could potentiate TE-1 cell proliferation, migration, and invasion by the endocrine mechanism. Obesity increased the expression of proproliferative and antiapoptotic genes, which contributed to the survival of TE-1. This study was a novel in vivo animal model of ESCC growth under the background of obesity by nude mice. Though nude mice are not prone to obesity, they are good candidates for the xenograft tumor, which make them suitable subjects for this study, and up to 35% (14/40) mice were chosen by the criterion as obese mice for further trials in the study. The model could also be used to further explore the mechanisms of how obesity affects ESCC growth. Tumorigenesis in obese patients is determined by a series of important mechanisms and metabolites such as insulin, insulin resistance, leptin, and visfatin [33, 34], while a strong positive correlation was observed between glucose, HOMA-IR, leptin, and visfatin in sera and xenograft tumor growth in this study. Despite these findings, it remains to be examined which specific adipose depots or obese effects are relevant to cancer growth and development.

Adipokines such as leptin and adiponectin are implicated in cell growth, proliferation, cell cycle control, and angiogenesis [12]; the results of this study also verified the relationship between ESCC growth and leptin. Among adipokines, growing interest has been placed in recent years on visfatin and its role in carcinogenesis. Visfatin may prompt tumor proliferation and metastasis in a number of cancers such as breast cancer [35], oral squamous cell carcinoma [36], and gastric cancer [37]. This study also proved the relationship between ESCC growth and visfatin. Visfatin is also functioning as a NAD biosynthetic enzyme which was overexpressed in multiple cancers including gastric cancer [38] and oral squamous cell carcinoma [39]. Another prominent adipokine, adiponectin, is mainly secreted from visceral AT but negatively related to adiposity, hyperinsulinemia, and inflammation. Adiponectin may exert anticancer effects by activation of AMPK [40]. AMPK is an important cellular energy sensor which is activated by increasing AMP levels. It works by coordinating cell growth with energy availability and is also inactivated in an obese state. Hence, the AMPK-YAP signaling pathway is closely related to the cellular energy state including glucose and lipid metabolism such as obesity and cancer. This study is the first to prove that obesity potentiated ESCC growth and invasion with relation to the AMPK-YAP signal pathway and reveal that obesity downregulated AMPK and p-AMPK, while upregulating MMP9, total YAP, p-YAP, and nonphosphorylated YAP protein expression. It is also found that obesity reduced AMPK and enhanced YAP and MMP9 expression in the mRNA level by complex effect including an important pathway, the endocrine mechanism.

IR was related to increased lung cancer risk [41], and insulin may also influence obesity-mediated tumors such as renal cancer and ovarian tumor [42, 43], but the relationship between insulin and ESCC growth had not been examined in the study. The novel findings of this study that serum visfatin and leptin were positively related to xenograft tumor growth were consistent with the notion that adipokines and other growth factors secreted on the background of obesity may prompt cancer cell survival and tumor growth. Certainly, limitations of the study existed; one of them is that nude mice are insensitive to establishing an obese model, and nude mice have only a small volume of serum unavailable for further analysis such as adiponectin and lipid assay. Another is that the complicated effect of obesity on the survival of mice bearing tumors could not be estimated.

## 5. Conclusion

IR and changed adipokine milieu observed in obesity may potentiate ESCC xenograft tumor growth and promote ESCC cell proliferation, migration, and invasion in vitro.

## Figures and Tables

**Table 1:** Sequences of the primers in the RT-PCR assay.

Genes	Primer	5′ → 3′ sequences	Product size (bp)
MMP9	Forward	CGCTGGGCTTAGATCATTCC	107
	Reverse	GTGCCGGATGCCATTCAC	
AMPK	Forward	GTAGTAAAAACAGGCTCCACGAA	76
	Reverse	CACCAGAAAGGATCTGTTGGA	
YAP	Forward	CGCTCTTCAACGCCGTCA	131
	Reverse	AGTACTGGCCTGTCGGGAGT	
GAPDH	Forward	ACTCCACGACGTACTCAGCG	224
	Reverse	GGTCGGACTCAACGGATTTG	

**Table 2:** The weight of body, liver, and their subcutaneous tumors was analyzed.

Parameters	Obese	Lean	Nonobese
Body weight (g)	30.18 ± 0.67^∗^	26.02 ± 2.32^∗∗^	25.15 ± 2.35
Liver weight (g)	1.03 ± 0.09^∗^	0.79 ± 0.08^∗∗^	0.81 ± 0.08
Tumor weight (g)	0.44 ± 0.25^∗^	0.18 ± 0.15^∗∗^	0.16 ± 0.14

^∗^ *P* < 0.05 versus lean and nonobese, ^∗∗^*P* > 0.05 versus nonobese.

**Table 3:** Serum metabolic and adipokine changes in mice.

Parameters	Groups		
	Obese	Lean	Nonobese
Insulin (ng/mL)	0.51 ± 0.05^∗^	0.43 ± 0.04	0.42 ± 0.05^∗∗^
Glucose (mmol/L)	11.4 ± 0.77^∗^	8.13 ± 0.76	7.65 ± 0.84^∗∗^
HOMA-IR	5.49 ± 0.61^∗^	3.28 ± 0.45	3.02 ± 0.52^∗∗^
Leptin (ng/mL)	0.25 ± 0.04^∗^	0.18 ± 0.03	0.17 ± 0.02^∗∗^
Visfatin (ng/mL)	90.55 ± 3.83^∗^	73.22 ± 10.54	74.47 ± 6.02^∗∗^

^∗^ *P* < 0.05 versus lean and nonobese, ^∗∗^*P* > 0.05 versus lean.

## Data Availability

The raw data of correlation analysis between serum glucose, insulin, HOMA-IR, leptin, visfatin, body weight, and liver weight of mice and weight of xenograft tumors which was not shown in the manuscript is available from the corresponding authors by request. All of the other data is provided within the manuscript.
